# Clinical outcomes associated with catecholamine use in patients diagnosed with Takotsubo cardiomyopathy

**DOI:** 10.1186/s12872-018-0784-6

**Published:** 2018-03-20

**Authors:** Uzair Ansari, Ibrahim El-Battrawy, Christian Fastner, Michael Behnes, Katherine Sattler, Aydin Huseynov, Stefan Baumann, Erol Tülümen, Martin Borggrefe, Ibrahim Akin

**Affiliations:** 1 First Department of Medicine, University Medical Center Mannheim, University of Heidelberg, Mannheim, Germany; 2 DZHK (German Center for Cardiovascular Research) partner site Mannheim, Mannheim, Germany; 3 First Department of Medicine, University Medical Center Mannheim, Theodor-Kutzer-Ufer 1-3, 68167 Mannheim, Germany

**Keywords:** Takotsubo cardiomyopathy, Cardiogenic shock, Heart failure, Catecholamines

## Abstract

**Background:**

Recent hypotheses have suggested the pathophysiological role of catecholamines in the evolution of the Takotsubo syndrome (TTS). The extent of cardiac and circulatory compromise dictates the use of some form of supportive therapy. This study was designed to investigate the clinical outcomes associated with catecholamine use in TTS patients.

**Methods:**

Our institutional database constituted a collective of 114 patients diagnosed with TTS between 2003 and 2015. The study-patients were subsequently classified into two groups based on the need for catecholamine support during hospital stay (catecholamine group *n* = 93; 81%, non-catecholamine group = 21; 19%). The primary end-point of our study was all-cause mortality.

**Results:**

Patients receiving catecholamine support showed higher grades of circulatory and cardiac compromise (left ventricular ejection fraction (LVEF) 39.6% vs. 32.7%, *p*-value < 0.01) and the course of disease was often complicated by the occurrence of different TTS-associated complications. The in-hospital mortality (3.2% vs. 28.5%, *p* < 0.01), 30-day mortality (17.2% vs. 51.4%, *p* < 0.01) as well as long-term mortality (38.7% vs. 80.9%, *p* < 0.01) was significantly higher in the group of patients receiving catecholamine support. A multivariate Cox regression analysis attributed EF ≤ 35% (HR 3.6, 95% CI 1.6–8.1; *p* < 0.01) and use of positive inotropic agents (HR 2.2, 95% CI 1.0–4.8; *p* 0.04) as independent predictors of the adverse outcome.

**Conclusion:**

Rates of in-hospital events and short- as well as long-term mortality were significantly higher in TTS patients receiving catecholamine support as compared to the other study-patients. These results need further evaluation in pre-clinical and clinical trials to determine if external catecholamines contribute to an adverse clinical outcome already compromised by the initial insult.

## Background

The Takotsubo Syndrome (TTS) is as an acute and usually reversible from of heart failure characterized by a transient dysfunction of the left ventricle [1, 2]. The TTS patient presents with clinical features such as acute chest pain and dyspnea, bearing some similarity to the patient presenting with an acute myocardial infarction or an acute coronary syndrome (ACS) [3]. An increase in cardiac troponin and creatine kinase levels as well as electrocardiogram (ECG) changes on admission suggesting ST-segment elevation in precordial leads adds to the confusion in early diagnosis. The absence of significant coronary stenosis on coronary angiography, which could correlate with this presentation, and the history of an emotional or physical trigger, leads to the potential diagnosis of TTS [3, 4]. This non-ischemic syndrome has also alternatively been labeled as a stress- or stress-induced cardiomyopathy, an apical ballooning syndrome, or ‘broken heart syndrome’ [4–8].

The classical pattern of left ventricular (LV) morphology in TTS, present in almost 50–80% of all patients, is the apical variant, which is characterized by apical ballooning of the LV at end-systole. Other morphological presentations include those defined by a predominantly hypokinetic circumferential base (inverted Takotsubo variant), or a hypokinetic circumferential mid ventricle (mid LV variant), or demonstrating focal variations [9–13].

The pathophysiology of TTS has been well-debated, however is still poorly described. The influence of an acute catecholaminergic surge contributing to some form of myocardial stunning has been hypothesized by several researchers [14]. Stressful triggers could potentially contribute to an increased hypothalamic-pituitary-adrenal axis (HPA) gain and catecholamine release. This resulting catecholamine surge possibly directs a pathological response from the cardiovascular as well the sympathetic nervous system and serves as the basis for the evolution of TTS [15, 16].

This interesting correlate, naturally, has far-reaching clinical implications. The therapeutic use of catecholamines has been routinely advocated in cases of circulatory compromise, however, its use in the setting of TTS could have potential drawbacks. Extrapolating this thought, our study attempts to explore the hither to poorly understood pathophysiological mechanisms involved in the TTS and determine if external catecholamines contribute to an adverse clinical outcome already compromised by the initial insult.

## Methods

### Study design and population characteristics

This study incorporated a population subset derived from a patient collective diagnosed with TTS at the University Medical Centre Mannheim, Germany between January 2003 and September 2015. A total of 114 patients were included consecutively to this mono-centric and observational study designed for retrospective data analysis. All these patients were essentially diagnosed with the TTS on hospital admission, and their presenting features met the conditions set out by the modified Mayo Clinic Criteria [17]. These criteria essentially highlight the transient wall motion abnormality in the LV mid-segments with or without apical involvement; describe regional wall motion abnormalities that extend beyond a single epicardial vascular distribution; and define an event that occurs frequently, but not always in the wake of a successful trigger. Additionally, these salient criteria mandate the effective rule-out of occlusive coronary disease; focuses on the appearance of new ECG pathologies, which mimic ACS or modest elevations in cardiac troponin levels; and underlines the prerequisite absence of diseases like pheochromocytoma and myocarditis in the patient.

**Fig. 1:**
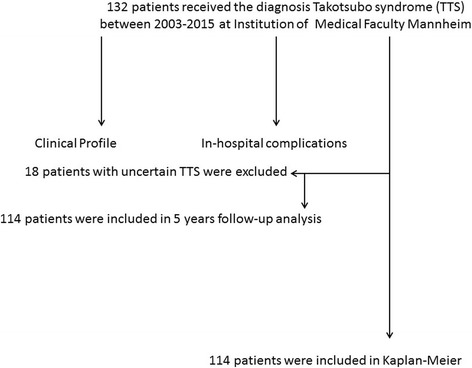
Flow Diagram of study

Patients with co-existing occlusive coronary artery disease as well as those exhibiting wall-motion abnormalities corresponding to any single coronary vessel territory were excluded from this study. A total of 18 patients with uncertain TTS, with no record of coronary angiography or echocardiography results, were excluded from this study. The relevant clinical data of each study-patient was ascertained and compiled in a database with significant aspects of their medical history, laboratory work-up and medical/surgical therapy efficiently earmarked for future reference. Additional parameters outlined for evaluation included duration of hospital stay, need for monitoring and care in the ICU (intensive care unit), use of invasive or non-invasive ventilation, inotropic support, temporary pacing, and demand for renal replacement therapy. Essential diagnostic workup including a routine ECG, echocardiography (to evaluate LV, RV function and wall-motion abnormalities), and a coronary and LV angiography (to rule out occlusive coronary artery disease) was performed on all patients. A consolidated review of this data was conducted by two independent cardiologists and once the diagnosis of TTS was reaffirmed, the study-patients were subsequently classified into two groups based on the need for catecholamine support during hospital stay. A consortium diagram to explain the population recruitment has been outlined in Fig. 1.

The primary end-point of our study was all-cause mortality as assessed by chart review and/or telephonic review. If medical records, treating physicians or relatives were unable to provide further information concerning the circumstances of death, it was defined as death due to unknown cause.

The research conduct corresponded to the principles outlined in the declaration of Helsinki and was approved by the medical ethics committee of the Faculty of Medicine in Mannheim, University of Heidelberg, Germany.

### Statistics

Statistical analyses were performed using SPSS Version 22 (SPSS Inc., Chicago, Illinois). Data is presented here as means ± SD for continuous variables with a normal distribution, median (interquartile range) for continuous variables with a non-normal distribution, and as frequency (%) for categorical variables. The Kolmogorov–Smirnov test was used to assess normal distribution. Student’s t-test and the Mann–Whitney U-test were used to compare continuous variables with normal and non-normal distributions, respectively. The chi-squared-test or Fisher’s exact test was used to compare categorical variables. The log-rank test was used to compare the survival curves between the two patient groups classified as per catecholamine use. *p*-values < 0.10 on univariate analysis were further evaluated via the Cox multivariate regression to define independent risk factors for the respective end-point. A two-tailed *p*-value of < 0.05 was considered statistically significant.

## Results

### Baseline characteristics

The baseline characteristics of the 114 patients included in this study have been referenced in Table 1.

**Table 1:** Baseline characteristics of 114 patients initially presenting with TTC

Variables	*No catecholamines* (*n* = 93)	*catecholamines*(*n* = 21)	*p* value*
Demographics			
Age, mean ± SD	67.65 ± 11.00	65.00 ± 12.30	0.33
Male, *n* (%)	13 (13.97)	6 (28.57)	0.11
Symptoms, *n* (%)			
Dyspnoe	34 (36.55)	9 (42.85)	0.62
Chest pain	52 (55.91)	6 (28.57)	**0.03**
Clinic parameter			
Systolic BP, mmHg	134.96 ± 28.41	116.25 ± 42.32	**0.01**
Diastolic BP, mmHg	78.41 ± 13.67	66.55 ± 31.22	**0.03**
Heart rate, bpm	98.17 ± 27.64	110.95 ± 22.79	**0.05**
ECG Data, *n* (%)			
ST-segment elevation	27 (29.03)	7 (33.33)	0.79
Inversed T-Waves	83 (89.24)	19 (90.47)	0.74
PQ-interval	160.67 ± 27.97	160.00 ± 34.38	0.92
QTc (ms), mean ± SD	484.49 ± 50.83	455.30 ± 52.82	**0.02**
Stress factor, *n* (%)			
Emotional sress	28 (30.10)	2 (9.52)	**0.05**
Physical stress	50 (53.76)	14 (66.66)	0.33
Laboratory values, mean ± SD			
Troponin I (U/L)	3.20 ± 4.41	6.57 ± 8.46	**0.01**
Creatine phosphatkinase (U/L)	721.11 ± 2900.64	323.74 ± 469.71	0.55
CKMB	37.44 ± 65.08	31.00 ± 23.56	0.77
C-Reactive protein (mg/l)	42.63 ± 64.25	80.30 ± 126.15	0.06
Hemoglobin	12.10 ± 1.99	12.29 ± 2.08	0.70
Creatinine (mg/dl)	1.14 ± 0.76	1.19 ± 0.50	0.79
GFR < 60 ml/min	25 (26.88)	7 (33.33)	0.59
Echocardiography data, *n* (%)			
LV EF %	39.67 ± 9.12	32.76 ± 9.09	**< 0.01**
Follow-up LV EF %	55.23 ± 7.54	48.63 ± 14.90	**< 0.01**
Apical ballooning	64 (68.81)	18 (85.71)	0.15
Mitral regurgation	50 (53.76)	10 (47.61)	0.63
Tricspid regurgation	42 (45.16)	7 (33.33)	0.46
RV-Involvement	17 (18.27)	9 (42.85)	**0.02**
Medical history, *n* (%)			
Smoking	30 (32.25)	6 (28.57)	0.80
Diabetes mellitus	22 (23.65)	4 (19.04)	0.64
Obesity (BMI > 25 kg/m^2^)	27 (29.03)	4 (19.04)	0.75
Hypertension	54 (58.06)	12 (57.14)	1.00
COPD	19 (20.43)	7 (33.33)	0.25
Atrial fibrillation	15 (16.12)	6 (28.57)	0.21
Coronary artery disease	16 (17.20)	6 (28.57)	0.23
History of malignancy	13 (13.97)	3 (14.28)	0.97
Drugs on admission, *n* (%)			
Beta-blocker	32 (34.40)	3 (14.28)	0.06
ACE inhibitor	30 (32.25)	5 (23.80)	0.45
ASS	24 (25.80)	5 (23.80)	0.87
Anticoagulation	7 (7.52)	0 (0)	0.19

*p values for the comparison between *group 1* and *group 2*; *SD* Standard deviation, *ECG* Electrocardiogram, *EF* Ejection fraction, *BMI* body-mass-index, *COPD* Chronic obstructive pulmonary disease, *ACE* Angiotensin-convetring-enzyme

EFhochversusnichthoch*Katecholaminpflichtigkeit: 0.000

The bolded indication highlight significant values

A detailed analysis of available data revealed an insignificant demographic distribution, with age and gender variables expressed almost similarly in both patient groups. Chest pain was interestingly more pronounced in the hemodynamically stable patient (55.9%, *n* = 52, vs. 28.5%, *n* = 6, *p* = 0.03) as compared to those requiring some form of catecholamine support. The clinical parameters used to ascertain patient status, such as systolic blood pressure (134.96 ± 28.41 mmHg vs. 116.25 ± 42.3 mmHg, *p* = 0.01) and heart rate (98.1 ± 27.6 bpm vs. 110.9 ± 22.7 bpm, *p* = 0.03), expectedly showed variation between the two groups, wherein lower blood pressure values and mild tachycardia was frequently observed among patients requiring catecholamine support.

### Diagnostic parameters

The initial diagnostic work-up, detailed with an electrocardiogram, suggested catecholamine-support-free patients recorded significantly longer QTc-Interval’s as opposed to the hemodynamically unstable catecholamine therapy-dependent patient (484.49 ± 50.83 ms vs. 455.30 ± 52.82 ms, *p* = 0.02). Other diagnostic parameters like echocardiographic estimates of left-ventricular ejection fraction revealed a comparatively lower degree of compromise in cardiac function among patients not requiring external catecholamines (index LVEF measurements 39.67 ± 9.12% vs. 32.76 ± 9.09%, *p* < 0.01). Similarly, lower index troponin-I levels were documented in this group (3.20 ± 4.41 U/l vs. 6.57 ± 8.46 U/l, *p* = 0.01). Right ventricular involvement was less pronounced in the non-catecholamine support patient-group (18.2%, *n* = 17 vs. 42.85%, *n* = 9, *p* = 0.02).

### In-hospital outcome

Data detailing in-hospital events and treatment strategies adopted for our study population suggested that patients suffering from hemodynamic compromise and requiring catecholamine support had a significantly increased incidence of life-threatening arrhythmias (33.3%, *n* = 7 vs. 6.4%, *n* = 6, *p* < 0.01) as well as cardiogenic shock (85.7%, *n* = 18 vs. 4.3%, *n* = 4, *p* < 0.01) and often required admission to an intensive care unit with longer stays (9.5 ± 11.3 days vs. 3.2 ± 3.5 days, *p* < 0.01). Rates of in-hospital death were higher among patients receiving catecholamine support (28.5%, n = 6 vs. 3.2%, *n* = 3, *p* < 0.01). Non-invasive positive pressure ventilation, endotracheal intubation and cardiopulmonary resuscitation procedures were practised significantly more often in patients constituting this group, Table 2.

**Table 2:** In-hospital events and treatment strategy

Variables	*No catecholamines* (*n* = 93)	*catecholamines* (*n* = 21)	*p* value*
Life-threatening arrhythmia	6 (6.45)	7 (33.33)	**< 0.01**
NPPV and intubation	21 (22.5)	18 (85.7)	**< 0.01**
Resuscitation	3 (3.22)	6 (28.57)	**< 0.01**
Defibrillator-Implantation	1 (1.0)	1 (4.7)	0.33
VA-ECMO	0 (0)	1 (4.7)	0.18
Admission to ICU, length of stay	3.24 ± 3.59	9.57 ± 11.32	**< 0.01**
In-hospital death	3 (3.2)	6 (28.5)	**< 0.01**
Thromboembolic events	10 (10.75)	4 (19.04)	0.29
Acquired Long QTs	61 (65.59)	12 (57.14)	0.41
Cardiogenic Shock	4 (4.30)	18 (85.71)	**< 0.01**

*p values for the comparison between *no catecholamines* and catecholamines; *NPPV* Noninvasive positive pressure ventilation, *VA-ECMO* Veno-arterial extracorporal membrane oxygenation, *ICU* Intermediate care unit

The bolded indication highlight significant values

### Long-term follow-up

**Fig. 2:**
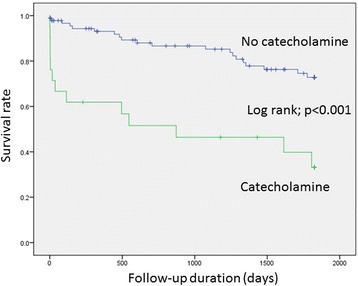
Log-rank and survival rates of patients treated and not treated with catecholamines

TTS patients receiving catecholamine support registered significantly higher 30-day mortality rates as compared to the clinically stable patient (57.14%, *n* = 12 vs.17.2%, *n* = 16, *p* < 0.01). Furthermore, these patients showed an ongoing increased risk of death beyond the first 30-days after hospital admission resulting in considerably higher long-term mortality-rates (80.95%, *n* = 17 vs. 38.70%, *n* = 36, *p* < 0.01); Table 3, Fig. 2. In a Cox univariate analysis EF ≤35% (HR 4.8, 95% CI 2.2–104; *p* < 0.01), male gender (HR 2.6, 95% CI 1.2–5.7; p 0.01) and use of positive inotropic agents (HR 3.9, 95% CI 1.9–7.9; *p* < 0.01) were associated with an adverse outcome. In comparison, a multivariate Cox regression analysis attributed EF ≤ 35% (HR 3.6, 95% CI 1.6–8.1; *p* < 0.01) and use of positive inotropic agents (HR 2.2, 95% CI 1.0–4.8; *p* 0.04) as independent predictors of the adverse outcome, Table 4. The various causes of death have been illustrated in Table 5.

**Table 3:** Outcome (mortality) in TTS patients

Variables	*No catecholamines* (*n* = 93)	*catecholamines* (*n* = 21)	Relative risk (95% CI)	*p* value *
In-hospital mortality	3 (3.22)	6 (28.57)	(1.0–6.5)	< 0.01
30-day mortality	3 (2.15)	6 (28.57)	(1.0–6.5)	< 0.01
1-year mortality	6 (6.45)	8 (38.09)	(1.1–3.7)	< 0.01
2-year mortality	11 (11.82)	10 (47.61)	(1.1–2.5)	< 0.01
3-year mortality	12 (12.90)	11 (52.38)	(1.1–2.5)	< 0.01
4-year mortality	18 (19.35)	11 (52.38)	(1.0–1.9)	< 0.01
Long-term mortality	20 (21.50)	13 (61.90)	(1.1–2.0)	< 0.01

*p values for the comparison between no catecholamines *and catecholamines*

**Table 4:** Multivariate analysis for the end point (all-cause mortality)

	Univariate analysis			Multivariate analysis^a^)		
	HR	95% CI	*P*-value	HR	95% CI	*P*-value
Male	2.6	1.2–5.7	**0.01**	1.8	0.8–4.0	0.14
EF ≤ 35%	4.8	2.2–104	**< 0.01**	3.6	1.6–8.1	**< 0.01**
Emotionalerstress	0.4	0.1–1.1	0.10			
Inotropic drugs	3.9	1.9–7.9	**< 0.01**	2.2	1.0–4.8	**0.04**
Diabetes mellitus Typ II	1.0	0.7–1.4	0.81			
Hypertension	0.9	0.7–1.2	0.64			
Apical ballooning	1.1	0.8–1.4	0.39			
History of cancer	1.7	0.7–4.2	0.21			
Smoking	0.7	0.3–1.6	0.49			

*HR* hazard ratio, *EF* ejection fraction*, CRP* c-reactive protein, *GFR* glomerular filtration rate

^a^Only the following variables with significant effects in univariate analysis were analyzed by multivariate Cox regression: Male, EF ≤ 35%, Inotropic drugs

The bolded indication highlight significant values

**Table 5:** All-Cause of death

In-hospital mortality	
1. Cardiac cause including cardiogenic shock and life-threatening arrhythmia (*n* = 6) 2. Malignoma (*n* = 1) 3. Neurological cause (*n* = 2)	
Out of hospital mortality	
1. Cardiac cause including cardiogenic shock and life-threatening arrhythmia (*n* = 4) 2. Malignoma (*n* = 6) 3. Sepsis (*n* = 4) 4. Kidney failure (*n* = 2) 5. Pulmonary cause (*n* = 2) 6. Unknown cause (*n* = 6)	

## Discussion

The principal foundation of this study was to determine the role and influence of catecholamine therapy in the treatment of the hemodynamically unstable TTS patient. At the outset, patients receiving catecholamine support showed higher degrees of circulatory and cardiac compromise, and the course of disease was often complicated by the occurrence of different TTS-associated complications. This group of patients also demonstrated poorer in-hospital outcomes and had higher long-term mortality rates as compared to the group of patients not receiving any catecholamine support therapy. It could therefore be inferred that circulatory support in the form of catecholamines entails a poorer outcome for the TTS patient. A pertinent question arises when debating the role of catecholamines exacerbating the mortality risk in such a clinical scenario solely on the basis of the pathophysiology of the syndrome. All patients, irrelevant of diagnosis, requiring catecholamine support constitute a clinically compromised group, however, do TTS patients in circulatory or cardiac shock suffer from additional drawbacks due to this line of management? Our results indicate that contemporary circulatory support drugs like catecholamines additionally exacerbates the risk of mortality in a TTS patient. This study is probably the first of many to follow, that has attempted to express this distinct correlation. Although, many studies have attributed some form of catecholamine toxicity leading to the pathophysiological evolution of TTS, we shall explore this line of discussion further, considering the potential impact on current treatment strategies.

A detailed research of recently published literature highlights the several hypotheses which have been postulated to explain the pathogenesis of TTS. These mechanisms essentially entail the possibility of coronary microvascular dysfunction, coronary artery spasm, catecholamine-induced myocardial stunning, acute LV outflow obstruction, acute increased ventricular afterload, myocardial microinfarction or abnormalities in cardiac fatty acid metabolism influencing the development of TTS [15].

A common link serving these several theories can be construed with the consistent demonstration of some form of microvascular dysfunction among all TTS patients. The research work conducted by Uchida et al. could successfully exhibit the presence of extensive endothelial apoptosis in myocardial biopsies [18] while Afonso et al. demonstrated circulatory disturbances in myocardial contrast echocardiography [19]. Abnormalities associated with endothelium dependent vasodilatation, excessive vasoconstriction and impairment of myocardial perfusion have been associated with coronary microvascular dysfunction seen in TTS patients [20]. The additional demonstration of regional contraction band necrosis, inflammatory cell inflammation and localised fibrosis, all attributed to direct catecholamine toxicity, further corroborates this theory [17, 21]. Interesting results interpreted from the research of Morel et al. suggested that an increase in C-reactive protein levels and white blood cell counts corresponded to increased levels of catecholamines in TTS patients [22]. The raised levels could in theory initiate a systemic inflammation mediated by cytokines like TNF-alpha and interleukin-6, explaining the observation of myocardial oedema in cardiac MRI [23]. In animal models of TTS, as prepared by Paur et al., the use of high bolus doses of catecholamines has simulated LV apical ballooning [24]. Additional studies by Wittstein et al., proving increased levels of circulating catecholamines in TTS patients as compared to those with myocardial infarction, as well as the theory of “stimulus trafficking” (involving a switch in intracellular signal trafficking from Gs protein to Gi protein) proposed by Lyon et al. have all given credence to the hypothesised pathogenic role of catecholamines in the development of TTS [24–26].

Although, these above-mentioned studies effectively hint at a definitive pathophysiological role played by catecholamines in the evolution of the syndrome, they fall short of explaining the potential effects of exogenous catecholamines in exacerbating a case of existing TTS.

A detailed analysis of our published research in this respect revealed that in-hospital events as well as short- and long-term mortality rates among TTS patients diagnosed with a significantly reduced LVEF on admission were significantly higher [27, 28]. We also showed that rates of in-hospital events and short- as well as long-term mortality rates were significantly higher in TTS patients suffering from arrhythmias such as atrial fibrillation [29, 30]. Although, such clinical scenarios do place the TTS patient at the worse end of the spectrum, it does not effectively explain the additional deleterious effects observed when patients received exogenous catecholamines as a form of therapy. An extrapolation of our data suggests reduced LVEF and use of positive inotropic agents as independent predictors of adverse outcome. A plausible explanation for this observation is perhaps the reactivation of catecholamine receptors or their downstream molecular pathways in the acute phase of TTS, which exacerbates the effects of the syndrome.

Our study has significant clinical implications. The essential role of catecholamines in the treatment of circulatory compromise has been consistently validated in the past, however, its use in TTS patients is now certainly questionable. Considering the pathophysiology of the syndrome, it would be pertinent to avoid any sort of catecholamine therapy for such patients. The Heart Failure Association of the European Society of Cardiology has recommended the avoidance or withdrawal of exogenous catecholamines as they could probably prolong or exacerbate the acute phase of the syndrome by activation of catecholamine receptors or their downstream molecular pathways [31]. It is for this reason that experts have suggested the early use of left ventricular assist device (LVAD) or extracorporeal membrane oxygenation (ECMO) as a bridge to recovery in highly unstable patients. Although treatment with levosimendan in this scenario is controversial, the absence of mechanical support may necessitate its use in certain scenarios [15].

This study clearly highlights the pitfalls associated with catecholamine use in patients suffering from circulatory compromise. The observation that most patients did not noticeably profit from this line of management and consistently suffered from poorer clinical outcomes with higher long-term mortality rates cements further support to recently postulated hypotheses and current recommendations for clinical management.

### Study limitations

This study was limited by its single-center retrospective observational study design, which included patients admitted over the period of 13 years. There was insufficient data detailing the proposed limits and quantified catecholamine doses used in these patients, furthermore, preventing the definitive assessment of the prognostic impact of different treatment strategies such as ECMO and intra-aortic balloon pump (IABP).

## Conclusion

This study equivocally suggests that TTS patients receiving catecholamine therapy show higher grades of circulatory and cardiac compromise, have poorer in-hospital outcomes and demonstrate higher long-term mortality rates in comparison to patients not receiving any form of catecholamine support. Although, these patients could in effect be suffering from a severe form of TTS, the interplay between the several mechanisms involved in the pathogenesis of this syndrome dictates the planning of therapeutic strategies precluding exogenous catecholamine use.

## Abbreviations

ACS: Acute Coronary Syndrome
ECMO: Extracorporeal membrane oxygenation
HPA: Hypothalamic-pituitary-adrenal axis
IABP: Intra-aortic balloon pump
ICU: Intensive care unit
LV: Left ventricle
LVAD: Left ventricular assist device
LVEF: Left ventricular ejection fraction
RV: Right ventricle
TTS: Takotsubo Syndrome
